# Low-carbohydrate ketogenic diet in Mc Ardle’s disease: a single-blinded randomized controlled trial

**DOI:** 10.1007/s00415-025-13405-5

**Published:** 2025-10-15

**Authors:** A. Martinuzzi, O. Musumeci, C. Stefan, E. Vinante, A. Ferrati, C. Perillo, N. Pesenti, A. Toscano

**Affiliations:** 1 Department of Public Health and Pediatric SCiences, Turin University, Turin, Italy; 2 Unit of Neurology and Neuromuscular Disorders, Department of Clinical and Experimental Medicine, University of Messina, Messina, Italy; 3 ERN-NMD Center for Neuromuscular Disorders of Messina, Department of Clinical and Experimental Medicine, AOU G. Martino of Messina, Messina, Italy; 4 Department of Conegliano, IRCCS E. Medea Scientific Institute, Conegliano, Italy

**Keywords:** Glycogenosis type 5, McArdle’s disease, Ketogenic diet, Clinical trial

## Abstract

Glycogenosis type 5 (GSD5, Mc Ardle’s disease), the most common muscle glycogen storage disease characterized by exercise intolerance, recurrent myoglobinuria and possible myopathic evolution, is still missing and effective treatment. A dietetic approach to circumvent the metabolic failure in GSD5 seems attractive and has been attempted in various forms but with mixed results. We ran a single-blind randomized controlled trial comparing 6 months of individualized 3:1 low-carbohydrate ketogenic diet (LCKD) to diet as usual in 21 adult patients with GSD5. Primary endpoints were safety and tolerability, and primary outcome was change in peak VO_2_ at incremental exercise test. Secondary outcomes were distance at the12minWT, change in peak work, changes in QoL measured by the SF36 and disability assessed by the WHO-DAS 2.0. Ketosis was monitored by checking blood levels of BOHB with portable glucometers. The LCKD regimen was well tolerated with no dropouts, no SAE related to treatment and only minor and transient subjective adverse events. Blood metabolites remained within the normal range. Six months of LCKD were associated with a significant increase in peak VO_2_ (+ 2.7 ml/min/Kg, *p* 0.049) and distance covered by the 12minWT (+ 55 m *p* 0.049). Peak work showed a marginal increase. There was a nonsignificant trend toward better perceived QoL and decreased disability by SF36 and WHO-DAS 2.0. LCKD is a safe and, once properly individualized, sustainable strategy to improve functioning in GSD5. Five patients in the LCKD study group elected to stay on LCKD and after 3 years are reporting good impact on functioning.

*Trial registration:* n°: NCT04292938, 3/25/2019.

## Introduction

McArdle’s disease (myophosphorylase deficiency, glycogen storage disease type 5, GSD5, OMIM # 232,600) is an inherited metabolic disorder of skeletal muscle. Affected patients suffer from genetically determined [1] lack of the enzyme muscle glycogen phosphorylase, which is essential for glycogen metabolism [2, 3]. The condition is caused by homozygous or compound heterozygous mutations in the muscle glycogen phosphorylase gene (*PYGM*) located at chromosome 11q13 [4]. Many pathogenic mutations have been identified in the gene, which spans 20 exons, and many are population specific [5, 6]. The most common mutation in Northern Europe and North America is a nonsense mutation at Arg50stop (R50X) in exon 1 (previously referred to as R49X) [7]. A second frequent mutation in this population and in Spanish patients is Gly205Ser (G205S) [6]. GSD5 is a rare disorder with an estimated incidence of 1:100,000 [8].

Complete absence of muscle phosphorylase results in the inability to mobilize muscle glycogen stores, normally required as substrate for energy generation during anaerobic metabolism, which occurs during start of exercise and high-intensity efforts. In affected people, symptoms of fatigue and discomfort therefore occur within minutes of initiating any activity and during strenuous activity such as lifting heavy weights or walking uphill. If the activity is continued despite symptoms, a severe contracture occurs, which leads to muscle damage. If the damage is substantial, acute rhabdomyolysis will ensue, which in turn can result in dark brown/black discoloration of urine (myoglobinuria). When rhabdomyolysis is severe, myoglobinuria can lead to acute renal failure, requiring treatment with dialysis.

In patients with GSD5, aerobic metabolism is limited and varies as a function of the availability of alternative fuels modulated by exercise and diet. The second wind phenomenon is illustrative. The phenomenon is characterized by the ability to increase work output after about 7–8 min of exercise. The second wind occurs as a consequence of increased availability and metabolism of alternative fuel substrates, preferentially glucose supplied from the liver, but also free fatty acids metabolized through oxidative phosphorylation and ketones produced by the liver. Despite these compensatory fuels, which can partially substitute for the absent glycogen breakdown in muscle, the capacity for oxidative phosphorylation is impaired in GSD5, because of an almost complete absence of pyruvate, a by-product of glycolysis [9–11].

Reduced oxidative phosphorylation in untrained patients with GSD5 in turn reduces oxygen consumption to approximately 35% of normal, and there is a disproportionate increase in heart rate during exercise in patients compared with healthy controls [12]. Thus, unconditioned people with GSD5 have very limited exercise capacity [13], which significantly affects their quality of life.

Most patients present in the second or third decade, although symptoms are often reported retrospectively from childhood. With advancing age, 20–25% of patients develop fixed muscle weakness predominantly affecting the shoulder girdle [13]. No clear-cut genotype–phenotype correlation has been found to explain the clinical variation in severity observed even within families, but the influence of polymorphisms in other genes has been hypothesized [14].

Currently, there is no treatment for the condition. There has been a small number of randomized controlled treatment trials, but the largest number of participants in any published study was 19 [15–17].

Taking glucose prior to exercise alleviates muscle symptoms by inducing a ‘second wind’ at the onset of exercise but has detrimental effects on weight if used too frequently [18, 19]. A Cochrane systematic review of training in GSD5 identified a few non-randomized trials of aerobic training or dietary manipulation either with supplements such as creatine or with shift toward lipid sources, which showed no harmful effect and suggested benefit over several months. However, long-term results and confirmation on larger cohorts are warranted [17].

Despite these indications, regular controlled training and dietary indications are seldom followed by patients, who experience significant limitations in activity of daily living and restriction in their participation.

A key limitation to exercise in GSD5 is the bottleneck in fuel flow through the tri-carboxylic acid (TCA) cycle, which is imposed by the minimal supply of glucosyl units from muscle glycogen and thus glycolytic flux to feed the TCA cycle [9, 20].

Dietary manipulation has been identified since the eighties as a potential strategy to improve functioning in GSD5 [21]. Despite initial indications for high-protein regimens, later experimental comparison of high-protein vs high-carbohydrate diets indicated a superiority for the latter [22]. Particular interest was also focused on diets with predominant lipid energy source (ketogenic or low-carbohydrate ketogenic: LCKD) with the assumption that ketones are easily taken up by mitochondria and can substitute for the missing acyl-CoA moieties not provided by the staggering glycolysis blocked upstream for the inaccessibility to muscle glycogen.

The strategy of modifying energy sources to improve functioning in GSD5 has been tested by using triheptanoine, an anaplerotic supplement providing an alternative source of Acetyl CoA and possibly increasing the succynil-CoA availability for the TCA, but the treatment failed to improve the exercise performance [23].

LCKD has a long history as a therapeutic strategy for several conditions (epilepsy, PDH defect, *GLUT1* mutations) with a good record of safety and efficacy and a poorer record of tolerability [24, 25]. Isolated experiences of LCKD have been carried out in GSD5 patients with promising results [26, 27]. A pilot study exploring acceptability and efficacy of few weeks of three different regimens of LCKD in a small number (eight) of GSD5 patients showed positive results, encouraging further exploration of this strategy [28]. The exploratory study was preparatory to a randomized single-blind placebo controlled cross over trial in which data were analyzed from 14 patients following 3 weeks of a modified ketogenic diet (75–80% fat, 15% protein, 5–10% carbohydrates) or placebo diet [29]. The trial failed its primary endpoint (heart rate change) but reported positive effects on patient-reported outcomes (SF36 and perceived exertion), peak VO_2_ and workload.

Here we report the results of a single-blinded randomized controlled trial testing the effects of 6 months of LCKD in a group of molecularly defined GSD5 patients. The trial (clinicaltrial.gov NCT04292938) originally intended as a multicenter trial recruiting 30 patients, due to the limitations imposed by the COVID-19 emergency, was realized only at two sites and recruited two thirds of the expected subjects. Still despite these limitations, we wish to share the results, also in consideration of the high expectations of the patients’ community on this issue [30].

## Methods

### Study design

The trial was designed as a randomized single-blind controlled trial testing the safety, tolerability and efficacy of an individualized LCKD in GSD5. Half of the recruited subjects were randomly selected to the dietary regimen, while subjects in the control group followed their usual balanced diet. The evaluators were blinded to the diet followed by the examined patient. The study was approved by the competent ethics committee (CESC #0199536) and was conducted in accordance with the Declaration of Helsinki.

### Patients

Twenty-one adult GSD5 patients were screened. Disease severity was staged according to published criteria [15]. Inclusion criteria were molecularly defined GSD5, age > 18, ability to perform a cycle ergometer exercise test.

Exclusion criteria were pregnancy, medical condition preventing a LCKD regimen (CPT2 or acyl-CoA deficiency, liver heart or kidney failure, type 1 diabetes). Recruited patients signed the informed consent to the study participation and data treatment.

### Intervention

Dietary modification, including the use of supplements, with the aim of reaching a lipid/carbohydrate 3:1 ratio with a minimum 1 g/Kg/die in protein. Ketosis was assessed via BOHB detection in blood sample with an expected BHOB level between 1.5 and 4 mmol/l. The dietary regimen was personalized by the dietician in its components to maximize acceptability, but always respecting the target 3:1 goal ratio. Similarly, the controls underwent a dietary evaluation to assure their habitual diet was sufficiently balanced. No change to usual diet habits was necessary for this group.

### Endpoints

The primary safety endpoint was absence of severe adverse events, number and frequency of minor side effects as recorded by an ad hoc diary. Primary tolerability endpoint was the rate of dropouts from the intervention group and the retention rate (n° of patients keeping detectable BOHB blood levels). Primary efficacy endpoint was the improvement in the aerobic capacity as measured by peak VO_2_ at exercise testing. Secondary endpoints explored the effect of the LCKD regimen on functional measures related to effort (12mWT), well-being and daily activities (SF36 and WHO-DAS 2.0) [31, 32], as well as the biochemical markers of metabolism and muscle integrity. The choice of these endpoints including a measurable biomarker, a performance outcome measure and two patient-reported outcome measures aimed at covering as much as possible the various domains of functioning with different approaches.

### Evaluations

All enrolled patients were examined at recruitment for BMI and blood test for CK, BUN, ammonia, uric acid, sugar, LDL/HDL cholesterol, triglycerides, FFA, ALT, AST, γGT, Na, K and Cl which were run.

All patients filled out a dietary questionnaire reflecting their actual dietary customs and preferences and performed a standardized cycle ergometer exercise test in which peak VO_2_, maximal workload and maximal heart rate were recorded (K5 metabolometer, Cosmed, Rome, Italy).

Exercise testing: Cycling exercise (20 min at 65% VO_2_ max followed by increments of 5 W per minute to exhaustion) was run on a cycle ergometer. The subjects wear a mask covering mouth and nose during cycling to measure O_2_/CO_2_-gas exchange rates (VO_2_ and VCO_2_). Subjects’ maximal oxidative capacities (peak VO_2_ in ml/Kg/min), as well as metabolic equivalents (METs), heart rate (bpm, beats per minute) and workload (peak W), were recorded at peak exercise before termination of the test for exhaustion.

Thirty minutes prior to the cycling exercise test, all patients performed a 12-min walking test and filled the SF36 and the WHO-DAS 2.0 self-administered forms. SF36 and WHO-DAS 2.0 total and domain specific scores were computed.

At the end of the baseline examination, eligible patients were randomized 1:1 to LCKD regimen or to control, according to a computer-generated randomization list (produced using the Proc Plan in SAS version 9.4 statistical package).

Patients randomized to active treatment were interviewed by the dietician, and according to their energy needs and food preference, a LCKD was constructed and prescribed. The diet included whenever appropriate special ketogenic food supplements added to the dietary regimen to ease tolerability. An induction period of 15–20 days was incorporated in the dietary prescription. The final prescribed diet provided adequate caloric intake with low-carbohydrate content (< 50 g/die providing < 20% of energy intake), a guaranteed minimum protein content (> 1 g/Kg/die) and the remaining energy provided by fat.

A portable glucometer by which the patient was instructed to monitor BOHB and sugar blood level was given to each patient together with direct demonstration and instruction for its home use.

A diary for annotation of any adverse event and a direct telephone number for signaling any problem was also given to each patient.

The patients were instructed to follow the prescribed diet for 6 months and to check BOHB levels daily for the first week, be-weakly for the second and third week, weakly for the remaining time.

Monthly prescribed diet to be eaten per person included as maximum carbohydrate content (as an example).

30 croissants, 1.5 kg pasta, 30 sandwiches/bread, 300 g breadsticks, 1 package of breadcrumbs, 1 flat bread (piadina). The remaining energy was provided by lipids, with care to assure the minimum protein content defined above.

Follow-up for clinical conditions, adverse events and adherence to protocol at week 12 were done by telephone. Safety and tolerability of the intervention were monitored by surveillance on any occurring severe adverse event (any event resulting in death or hospital admission) and by registration of all adverse reactions reported on a day-by-day basis by the patient during LCKD diet and during the post-diet follow-up lasting one month.

At sixth months (week 24), all the recruited patients repeated the evaluations performed at entry.

Patients who tested LCKD and found it sustainable and felt better were allowed to keep their preferred dietary regimen.

### Statistical analysis

Descriptive statistics were used to summarize the demographic and baseline clinical characteristics of the study population, with values reported separately for the two groups. The comparisons of these characteristics between the control and LCKD groups were assessed using either Student’s t-tests for continuous variables or Fisher’s exact test for categorical variables, as appropriate.

For the analysis of the primary and secondary outcomes, paired t-tests were performed within each group to compare the values at baseline (T0) and at six months (T6). To account for multiple comparisons, the resulting *p* values were adjusted using the false discovery rate (FDR) method. All statistical analyses were performed using R software (R Foundation for Statistical Computing, Vienna, Austria). Two-tailed tests were applied throughout, with *p* values less than 0.05 considered statistically significant.

## Results

Twenty-one patients were recruited, ten were assigned to the control group and eleven to the LCKD group. The demographics and clinical severity staging [14] of the recruited patients are provided in Table 1. The two groups were similar for age, BMI and disease severity. Males were nonsignificantly more numerous in the control group. The *PYGM* genotype of each patient is reported in Table 2. Baseline evaluations (Table 3) were similar between the two groups for all measures with the exception of the 12-min WT and maximal work that were significantly lower in the LCKD group. Similarly, disease perception and disability were significantly more severe in the treatment group. No significant difference was recorded in any of the biochemical blood tests. Two patients, belonging to the control group, dropped out of the study due to non-compliance with the programmed assessments. One patient in the treatment group while on trial discovered a gynecological malignancy and she was excluded from the final evaluation. Thus, the total cohort completing the study consisted of eighteen subjects, ten in the LCKD group and eight in the control group.

**Table 1:** Demographic characteristics of the population

Characteristics		LCKD (*N* = 11)	Controls (*N* = 10)	*p* value
Age, years—mean (SD)		43.09 (13.59)	45.30 (12.65)	0.705*
BMI, kg/m^2^—mean (SD)		24.9 (4.5)	25.8 (4.0)	0.645*
Sex, female—*n* (%)		7 (63.6)	2 (20.0)	0.081°
Disease Severity—*n* (%)	1	9 (81.8)	6 (66.7)	0.770°
	2	2 (18.2)	2 (22.2)	
	3	0 (0.0)	1 (11.1)	

Disease severity categorized according to published criteria [14]. *p* value from: * *t* test, ° Fisher exact test

**Table 2:** *PYGM* mutations in the two groups

Patient ID	LCKD (*N* = 11)	Controls (*N* = 10)
1	G > A c.2422–1/p.Tyr186*	
2	c.2262del/p.Arg428Cys	
3	p.Arg50*/p.Arg428Cys	
4	p.Arg50*/p.Arg50*	
5	p.Arg270*/IVS13 + 1G < A	
6	p.Arg50*/p.Arg50*	
7	p.Arg50*/1275 + 1G	
8	p.Arg50*/p.Arg50*	
9	c.660 + 703_660 + 766del/p.Arg50*	
10	p.Leu587Pro/p.Lys754Asn	
11	p.Lys754Asn/p.Lys754Asn	
12	p.Arg50*/p.Arg50*	
13		p.Arg50*/p.Ala704Val
14		p.Arg576*/p.Arg576*
15		p.Arg50*/p.Arg50*
16		p.Arg50*/p.Arg50*
17		p.Arg50*/pVal456Met
18		p.Arg428Cys/p.Leu397Pro
19		p.Arg428Cys/p.LeuPro
20		p.Leu587Pro/p.Thr379Me
21		p.Arg50*/p.Arg50*

**Table 3:** Baseline evaluations in the two groups; mean (SD)

Baseline evaluation	LCKD (*N* = 11)	Controls (*N* = 10)	*p* value
Peak VO_2_ ml/min/Kg	12.59 (2.78)	13.56 (3.62)	0.497
METs	3.61 (0.50)	4.04 (1.03)	0.312
Peak power W	47.82 (14.73)	70.30 (26.05)	0.023
Peak heart rate bpm	131.36 (39.05)	152.20 (20.90)	0.150
12mwt m	723.67 (174.73)	922.63 (128.98)	0.008
Cholesterol mg/dL	197.82 (41.70)	203.70 (23.85)	0.700
Triglycerides mg/dL	89.27 (25.73)	114.78 (79.05)	0.325
Proteins g/dL	7.25 (0.62)	7.08 (0.18)	0.506
CK IU/L	2505.2 (1959)	9700 (19,085)	0.227
Calcium mg/dL	6.52 (2.61)	6.38 (2.48)	0.904
Uric acid mg/dL	5.22 (1.08)	6.46 (1.99)	0.121
Vitamin D ng/ml	51.71 (18.03)	50.88 (12.00)	0.910
SF.36.Physical score	42.39 (23.18)	49.24 (16.67)	0.468
SF36.Mental score	58.18 (14.44)	72.43 (12.64)	0.027
WHO-DAS 2.0 score	29.20 (18.75)	11.03 (7.01)	0.019

*p* values from *t* test

The diet regimen was followed with some effort by all patients of the LCKD group and BOHB levels become measurable in all after 2 weeks of dieting. However, recorded levels were lower than expected ranging from 0.1 to 2.2 mmol/l (mean 1.07 ± 0.9). LCKD was well tolerated: No severe adverse event was recorded during the study, with the only exception of the above-mentioned malignancy, not considered related to treatment. The most frequently reported minor adverse event was nausea (14 times) followed by lethargy (9 times) constipation (7 times), loss of appetite (5 times) and diarrhea (2 times) (Table 4). All reported complaints resolved by the intermediate control at 3 months. Blood biochemical profile was not modified significantly in any parameter in both the treatment and the control groups. A nonsignificant increase in cholesterol was recorded in both groups and the uric acid content showed a mild increase in the LCKD group (+ 1 mg/dL *p* = 0.183) in T6 compared to T0. A summary of the most relevant measured hematochemical variables is shown in Table 5.

**Table 4:** Adverse events count: *n*° of total events reported by all patients

Adverse events	*n*° of events
Halitosis	0
Nausea	14
Vomit	0
Diarrhea	2
Constipation	7
Appetite loss	5
Lethargy	9

**Table 5:** Hematochemical variables assessed in two groups at T0 and T6

Metabolites		T0	T6	Adj *p* value
Cholesterol mg/dL	LCKD	195.9 (43.4)	210.3 (46.3)	0.312
	Control	197.6 (22.8)	208.6 (37.7)	0.939
Triglycerides mg/dL	LCKD	91.9 (25.5)	74.4 (26.7)	0.312
	Control	116.8 (84.3)	115.0 (49.8)	0.939
Proteins g/dL	LCKD	7.3 (0.7)	7.3 (0.6)	0.577
	Control	7.1 (0.2)	7.4 (0.3)	0.677
CK (IU/L)	LCKD	2728.6 (1911.4)	4003.3 (5068.8)	0.577
	Control	11,072.4 (21,727.4)	2585.0 (1897.4)	0.939
Calcium mg/dL	LCKD	6.3 (2.6)	6.2 (2.2)	0.843
	Control	6.8 (2.7)	5.9 (2.1)	0.939
Uric acid (mg/dL)	LCKD	5.4 (1.0)	6.4 (1.4)	0.183
	Control	6.5 (2.1)	6.9 (2.1)	0.939
Vitamin D ng/ml	LCKD	51.7 (18.0)	62.3 (17.1)	0.341
	Control	50.5 (9.5)	49.6 (18.5)	0.939

*p* value from: paired *t* test, multiple comparison adjustment: fdr

BMI decreased significantly in the treatment group and did not change in the control group.

Variations observed between T0 and T6 in the indicators of the exercise and walking performance are shown in Table 6. Peak VO_2_ showed a significant increase in the treatment group (+ 2.70 ml/Kg/min, *p* = 0.049), while it did not change in the control group (+ 0.6 ml/Kg/min, *p* = 0.939).METs significantly increased by 0.90 (*p* = 0.049) in the treatment group and remained unchanged in the control group. Maximal heart rate showed a significant increase after 6 months in the LCKD group (+ 14.6 bpm, *p* = 0.029) and remained substantially the same (+ 2.0 bpm) in the control group. Peak work increased marginally in the LCKD group (+ 1.9 W, *p* = 0.746) while it declined slightly in the control group (− 2.5 W, *p* = 0.939).

**Table 6:** Longitudinal variations in efficacy indicators of the two groups

Indicator		T0	T6	Adjusted *p* value
BMI: Kg/m^2^	LCKD	24.9 (4.5)	22.6 (3.4)	0.049
	Control	25.8 (4.0)	26.1 (4.3)	0.939
Peak VO_2_: ml/min/Kg	LCKD	12.3 (2.7)	15.0 (2.3)	0.049
	Control	14.1 (3.6)	14.7 (3.1)	0.939
METs	LCKD	3.5 (0.4)	4.4 (0.7)	0.049
	Control	4.3 (1.0)	4.3 (0.8)	0.939
Peak power: W	LCKD	46.8 (15.1)	48.7 (14.7)	0.746
	Control	76.4 (23.4)	73.9 (22.3)	0.939
Peak Heart rate: bpm	LCKD	132.3 (41.0)	146.9 (35.8)	0.029
	Control	158.0 (17.5)	161.9 (21.6)	0.939
12mwt: m	LCKD	718.3 (183.2)	773.3 (215.5)	0.049
	Control	953.6 (104.7)	909.0 (147.5)	0.837
SF_36_Physical score	LCKD	42.0 (24.4)	54.1 (24.3)	0.183
	Control	49.9 (14.7)	58.4 (16.4)	0.939
SF_36_Mental score	LCKD	57.5 (15.0)	64.8 (20.5)	0.287
	Control	74.7 (8.6)	73.0 (10.8)	0.939
WHO-DAS 2.0 score	LCKD	29.8 (19.6)	19.7 (17.1)	0.183
	Control	12.1 (6.8)	12.6 (16.8)	0.939

*p* value from: paired *t* test, multiple comparison adjustment: fdr

The LCKD group showed after 6 months of treatment a significant improvement of the 12-min WT (+ 55.0 m, *p* = 0.049) while the distance covered by the patients in the control group slightly decreased (− 44.7 m, *p* = 0.837).

Neither the SF36 nor the WHO-DAS 2.0 showed significant changes comparing T0–T6 scores in both groups. However, we recorded in the LCKD group a trend toward improved perception of physical (+ 12.1, *p* = 0.183) and mental (+ 7.4, *p* = 0.287) health and a decrease in experienced disability (− 10.2, *p* = 0.183). No changes were recorded in the control group.

## Discussion

We present the results of a controlled randomized trial comparing LCKD at 3:1 ratio with usual diet in a relatively small group of patients with GSD5. The trial, initially designed as involving three centers, was conducted at two centers with a reduced number of subjects due to the limitations related to the pandemics. This reduces the power of the study and limits its significance. Nevertheless, the study enrolled one of the largest GSD5 cohorts for a RCT and it conveys some very relevant messages. Safety, feasibility and tolerability of the dietary manipulation proposed in the study are demonstrated by the lack of dropouts for non-compliance with the diet and by the lack of worrisome modifications in blood tests. BOHB measurements certified the reached ketosis, even though not at the expected levels. The avid muscle uptake of ketones to feed the deprived TCA cycle could explain this observation, but more accurate metabolic study possibly with labeled metabolites could clarify this issue.

A side effect expected and welcomed by the patients is the significant reduction in BMI a frequently reported phenomenon in subjects following LCKD [33]. This leaning effect might be particularly appropriate in GSD5 patients that often are overweight [34].

The duration of the diet maintained in this study is among the longest documented for a monitored group of GSD5. Five of the ten subjects assigned to LCKD choose to continue the diet and at the last follow-up three years after starting the diet are still following it with reportedly very good effects on their functioning. One of the possible explanations for this good compliance could be due to the careful individualization of the dietary indications that, while guaranteeing the target nutrients ratio, allowed for adaptation to individual taste and preference. The primary efficacy end point was met: we observed a significant improvement in peak VO_2_. Similarly, the METs improved slightly but significantly, while the power increased only marginally.

More difficult to explain is the significant increase in maximal heart rate observed at T6 in the LCKD group.

Three weeks of LCKD diet in elite racewalkers resulted in a significantly higher heart rate [35], but given the specific significance of heart rate response to exercise in GSD5 patients, this issue deserves further consideration.

Our results reporting the significant increase in the peak VO_2_ are in accordance with the data reported by the Danish group for the short-term trial with modified ketogenic diet [29], although the different design of the exercise test makes the comparison difficult. Their recording of an unchanged peak heart rate, the primary chosen end point, led the researchers to conclude that the ketogenic regimen did not alleviate all the McArdle’s disease-related symptoms. However, given the short duration of the study, performance measures relevant for ADL, such as the 12mWT were not included. In our study the distance covered in the 12mwt significantly increased under LCKD, implying a parallel transfer of the benefit recorded with the exercise test to a typical performance outcome measure pertinent to activity of daily living. A 30-m increase in distance covered at the 6-min walking test, the more frequently used performance measure in neuromuscular diseases is considered clinically meaningful [36]. This positive impact on everyday life perceived by the patients emerges, even though nonsignificantly, from the results of the PROMs, the QoL and disability assessments, with improvement in the first and decrease in the second.

Limitations of the study are the smaller than planned number of recruited subjects and the lack of a careful assessment with labeled metabolites to fully document the metabolic shift and to positively prove the inferred improvement of the TCA flux. An additional limitation is the heterogeneity of the dietary regimens followed by the patients before entering the trial. This might have produced some bias. However, no strict dietary regimen was reported to the dietician by any patient during the recruitment evaluation, and all patients reported in their diary diets that were in substantial caloric and nutrients equilibrium compatible with a typical Mediterranean diet. We do not have results on the long-term effects on muscle structural changes that have been detected by MRI [37] in GSD5 subjects, but an observational study on subjects who choose to remain on LCKD diet could be our next endeavor, since all the subjects participating this trial have gone through an MRI study in the years preceding this study. Even considering these weaknesses, the study conveys an important message to the neurologists following GSD5 patients and to the patients’ community as well: A personalized dietary manipulation strictly limiting carbohydrates and favoring lipids in GSD5 is safe, feasible, sustainable and brings improvements in aerobic capacity translating into better motor performance in a daily routine task. Larger and longer studies should be encouraged by these results.

## Data Availability

Data will be provided upon resonable request to corresponding author.

## Abbreviations

GSD5: Glycogen storage disease type 5
LCKD: Low-carbohydrates ketogenic diet
VO_2_: Volume of oxygen consumed
12mWT: 12 Minutes walking test
QoL: Quality of life
SF36: Short form 36
WHO-DAS 2.0: WHO disability assessment schedule 2.0
SAE: Severe adverse event
TCA: Tri-carboxylic acid
CPT2: Carnitin palmitoyl tranferase 2
BOHB: β-Hydroxybutyrate
BMI: Body mass index
METs: Metabolic equivalent of task
Bpm: Beats per minute
ADL: Activities of daily living
PROMs: Patient-reported outcome measures
